# Sleep Disorders in Climacteric Women: Glutathione, Glutathione S-Transferase P1 and Gut Microbiome Interrelation

**DOI:** 10.3390/pathophysiology33010003

**Published:** 2025-12-26

**Authors:** Natalya Semenova, Nadezhda Garashchenko, Olga Nikitina, Sergey Kolesnikov, Natalia Belkova, Elizaveta Klimenko, Nadezhda Smurova, Elizaveta Novikova, Irina Madaeva, Liubov Kolesnikova

**Affiliations:** Scientific Centre for Family Health and Human Reproduction Problems, 664003 Irkutsk, Russia; nadzelin@mail.ru (N.G.); olga_tolpygina@mail.ru (O.N.); sikolesnikov1@rambler.ru (S.K.); nlbelkova@gmail.com (N.B.); klimenko.elizabet@gmail.com (E.K.); nadinasmurova@mail.ru (N.S.); yelizaveta_novikova_2001@bk.ru (E.N.); nightchild@mail.ru (I.M.); kolesnikova20121@mail.ru (L.K.)

**Keywords:** glutathione, glutathione S-transferase, gut microbiome, menopause, insomnia, sleep disorders

## Abstract

**Background**: Menopause, a critical period during a woman’s life, is characterized by various changes, including disturbances in their oxidative balance and circadian rhythm. Currently, the gut microbiome is suggested as an important participant in these processes. **Methods**: This study involved 96 menopausal women. Their sleep quality was assessed using three questionnaires: the Insomnia Severity Index (ISI), the Pittsburgh Sleep Quality Index (PSQI), and the Epworth Sleepiness Scale (ESS). The GSH and GSTP1 contents in the serum were measured by means of immunoassay methods, while the composition of the gut microbiome was determined via molecular genetic methods. **Results**: *E. coli*, *K. oxytoca*, *S. aureus*, *Enterobacter* spp., *Shigella* spp., *Streptococcus* spp., *Prevotella* spp., and *M. stadmanae* were found to correlate with the GSH content in different sleep groups, while the presence of *K. oxytoca*, *S. aureus*, *Enterococcus* spp., *K. pneumoniae*, and *M. stadmanae* is also important for the GSH level in several of these groups. *F. prausnitzii*, *S. aureus*, *P. micra*, *Acinetobacter* spp., and *E. rectale* are associated with GSTP1 concentration in various sleep groups, while the presence of *F. nucleatum* and *P. micra* is also relevant for the GSTP1 content in some of these groups. **Conclusions**: Thus, in menopausal women, the composition and structure of the gut microbiota are associated with sleep disorders. GSH and GSTP1 are associated with some gut microbiome markers in menopausal women, but these relationships differ in different sleep disorders.

## 1. Introduction

Sleep is suggested to have a protective role against oxidative damage due to its influence on the efficiency of the antioxidant defense system. Many results support this hypothesis [1,2]. In turn, insufficient sleep in terms of quality and/or longevity may impact a number of biochemical parameters associated with oxidative stress (OS) and inflammation [3]. Synthesized and maintained at high concentrations in all cells, the sulfhydryl-containing tripeptide glutathione (GSH) is a key protector against oxidative stress [4,5]. Glutathione transferase (GST) is also one of the most important antioxidant enzymes, along with glutathione peroxidase, glutathione reductase, superoxide dismutase, and catalase [6]. GST plays a major role in cellular redox-dependent processes, catalyzing the conjugation of GSH with non-polar compounds of endogenous and exogenous origin containing electrophilic atoms of sulfur, nitrogen, and phosphorus, as well as with organic hydroperoxides, and protecting the cell from the possible toxic effects of these compounds. GST includes three superfamilies: cytoplasmic, mitochondrial and microsomal. Cytoplasmic GST enzymes can fall into different classes of enzymes, among which GSTP1 is one of the most well studied and is most closely related to the development of human diseases [7,8].

Evidence exists for the interactions between the gut microbiome and OS; however, sleep disturbances could be a mediating factor in this relationship [9]. It is suggested that the gut microbiome regulates its host’s sleep through the gut–brain axis and is involved in sleep disorder development [10,11,12,13]. On the other hand, sleep deficiency apparently leads to microbiome alteration because of the accumulation of reactive oxygen species (ROS) in the gut, affecting bacteria directly or through the immune system [14].

The menopause is one of the most critical periods in a woman’s life, with sleep disorders being one of the main signs of the neurovegetative changes that occur during this process [15]. The menopause represents the termination of reproductive function and ovarian failure, with the decline in female sex hormone levels leading to various short- and long-term changes. Insomnia occurs in more than half of postmenopausal women [16]. Oxidative balance is also affected by menopausal changes, and it has been shown that postmenopausal women have higher levels of ROS compared with premenopausal women, indicating the development of oxidative stress [17,18,19]. Previous results have shown that various bacteria are involved in this process [20]. Meanwhile, the glutathione system is one of the most important participants in the processes maintaining the stability of molecular metabolic parameters [21].

The aim of this study was to reveal the correlations between GSH and GSTP1 levels and the gut microbiome in climacteric women with sleep disturbances.

## 2. Materials and Methods

### 2.1. Standard Protocol Approvals, Registrations, and Patient Consent

This study was conducted at in the Scientific Centre for Family Health and Human Reproduction Problems (Irkutsk, Russia). Informed consent was given by the participants in accordance with the Ethical Norms of the Declaration of Helsinki of the World Medical Association. The research protocol was approved by the Committee on Biomedical Ethics of the Scientific Centre (protocol No 3.1.3, dated 28 September 2022).

### 2.2. Subjects

In the first stage, 112 women were recruited. The primary inclusion criteria were a climacteric status and an age from 45 to 69. In addition, all women reported the presence of amenorrhea or menstrual irregularities, consisting of stable fluctuations (7 days and above) during successive cycles. The exclusion criteria were as follows: a regular menstrual cycle, an AMH level > 1 ng/mL, exacerbation of chronic diseases, diabetes mellitus, oncological disease, infectious diseases, and antibacterial medication in the past three months. Ultimately, the study sample included 96 volunteers. All participants provided written informed consent.

### 2.3. Methods

#### 2.3.1. Questionnaire

Grouping was based on the results of three self-administered questionnaires.

The Pittsburgh Sleep Quality Index (PSQI) consists of 19 items and is the most widely used retrospective self-report questionnaire assessing sleep quality over the previous month. The questionnaire analyzes seven sleep domains (sleep quality, sleep latency, sleep duration, sleep efficiency, sleep disturbances, sleep medication use, and daily activities). These domains are combined to create a single factor, global sleep quality. The results were assessed according to the standard protocol, with higher scores indicating poorer sleep quality. PSQI > 5 has been validated as a cut-off for sleep disturbances (SD group) across a number of populations [22,23].

The Insomnia Severity Index (ISI) is a reliable tool for quantifying the perceived severity of insomnia. The ISI is a 7-item self-report questionnaire that assesses the nature, severity, and impact of insomnia. The standard period is the “past month”, during which the following dimensions are assessed: difficulty initiating and maintaining sleep, difficulty waking early in the morning, sleep dissatisfaction, the impact of sleep disturbances on daytime functioning, the visibility of sleep problems to others, and stress caused by sleep difficulties. The ISI includes 7 items with a total score of 0–28. The classical interpretation according to the guidelines is as follows: control group < 8 points (N group); subthreshold insomnia 8–14 points (SubI group); clinical moderate insomnia 15–21 (I group); and severe insomnia > 21 (SevI group) [24]. However, since the last two groups are basically clinical insomnia groups, they were combined and a 3-group analysis was carried out. Binomial division is also possible, with a threshold equal to 10 suggested as the best option (ISI ≥ 10 indicates SD) [25].

The Epworth Sleepiness Scale (ESS) is a subjective measure of sleepiness that is widely used in clinical practice to identify and assess the severity of excessive daytime sleepiness. ESS is an 8-item questionnaire that asks patients about their likelihood of falling asleep in normal situations. ESS has a threshold > 10 (S group) [26,27]—this is not the only cut-off option, but is the most common one [28].

#### 2.3.2. Collection of Materials

Between 8.00 and 10.00 a.m., after 12 h of overnight fasting, venous blood was sampled from the cubital vein of each subject into a tube with a clot activator to obtain serum. Then, the samples were centrifuged for 10 min at 1500× *g*. The remaining blood serum was collected in an Eppendorf tube and frozen. Serum was used to assess the AMH, GSH, and GSTP1 concentrations. Also, each participant provided fresh stool samples on the same day. Feces were collected in test tubes with beads for material homogenization (BashingBead; Zymo Research, Tustin, CA, USA). All samples were stored at −80 °C prior to the assays being carried out.

#### 2.3.3. GSH

The GSH level (µg/mL) in serum was measured with the ELISA kit for Research Use Only “BLUE GENE” E01G0233 on “ELx808IU” analyzer (BioTek, Winooski, VT, USA) according to the manufacturer’s instructions.

#### 2.3.4. GSTP1

GSTP1 concentration (ng/mL) in serum was determined with the ELISA kit for Research Use Only “BLUE GENE” E01G0442 on “ELx808IU” analyzer (BioTek, Winooski, VT, USA) according to the manufacturer’s instructions.

#### 2.3.5. Gut Microbiome

The fecal DNA of each subject was extracted with the Stool Genomic DNA Kit (CWBIO, Beijing, China) according to the routine protocol. The quantitative assessment of the microbiocenosis state was investigated with RT-PCR, with fluorescent detection of amplification results using the “COLONOFLOR” reagent kit (Alphalab, Saint Petersburg, Russia) on a CFX-96 thermocycler (BioRad, San Mateo, CA, USA). Samples with concentrations ranging from 1 to 2 ng/μL were considered in this study. The Colonoflor-16 (premium) kit combines the Colonoflor-16 (biocenosis) and Colonoflor-16 (metabolism) reagent kits and allows for the quantitative (lg CFU/g) determination of the total bacterial mass, *Lactobacillus* spp., *Bifidobacterium* spp., *Bacteroides* spp., *Bacteroides thetaiotaomicron* (*B. thetaiotaomicron*), *Faecalibacterium prausnitzii* (*F. prau*), *Escherichia coli* (*E. coli*), *Akkermansia muciniphila* (*A. muciniphila*), *Enterococcus* spp., enteropathogenic *E. coli*, *Klebsiella pneumonia* (*K. pneumonia*), *Klebsiella oxytoca* (*K. oxytoca*), *Candida* spp., *Staphylococcus aureus* (*S. aureus*), *Clostridium difficile* (*C. difficile*), *Clostridium perfringens* (*C. perfringens*), *Proteus vulgaris/mirabilis* (*P. vulgaris/mirabilis*), *Citrobacter* spp., *Enterobacter* spp., *Fusobacterium nucleatum* (*F. nucleatum*), *Parvimonas micra* (*P. micra*), *Salmonella* spp., *Shigella* spp., *Blautia* spp., *Acinetobacter* spp., *Eubacterium rectale* (*E. rectale*), *Streptococcus* spp., *Roseburia inulinivorans* (*R. nulinivorans*), *Prevotella* spp., *Methanobrevibacter smithii* (*M. smithii*), *Methanosphaera stadmanae* (*M. stadmanae*), *Ruminococcus* spp., and the *Bacteroides* spp./*F. prau* ratio.

#### 2.3.6. Statistical Analysis

Statistical analysis was carried out with R Studio 2024.12.1 Build 563. The following libraries were used: “ggplot2”, “readxl”, “table1”, “boot”, and “dplyr”. Data in tables are presented as Me [Q1; Q2]. To calculate correlation, the Spearman rank correlation test was used, and 95% confidence intervals for Spearman correlations were obtained using the bootstrap method with 10,000 resamples. To compare two groups, the Wilcoxon rank sum exact test was applied. To compare several groups, the Kruskal–Wallis test was carried out. Subsequently, to identify groups with significant differences, the pairwise Wilcoxon rank test with Bonferroni correction was applied. Values of *p* < 0.05 (comparison of two groups), *p* < 0.017 (comparison of three groups), or *p* < 0.013 (comparison of four groups) were considered statistically significant.

## 3. Results

### 3.1. Characteristics of PSQI Groups

Based on PSQI scores, participants were categorized into two groups (Table S1, Supplementary Material). Three bacterial parameters differed significantly between these groups, namely, *Enterococcus* spp. (*p* = 0.025), *Shigella* spp. (*p* = 0.04), and *Clostridium perfringens* (*p* = 0.013), with the levels of these bacteria being higher in the group with sleep disorders. Moreover, the differences in *Shigella* spp. were significant, since their values in the group with poor sleep quality were above Q3.

### 3.2. Characteristics of ISI Groups

One method of analyzing the ISI questionnaire involves binomial grouping (Table S2, Supplementary Material). The total bacterial mass is lower in the insomniac sleep disorder group (*p* = 0.03). Additionally, significant differences were detected for *Clostridium perfringens* (*p* = 0.002), *Streptococcus* spp. (*p* = 0.03), and *Shigella* spp. (*p* = 0.03). The differences in *Shigella* spp. were significant, as their values in the group with poor sleep quality were above Q3, as in PSQI grouping.

The four-group ISI classification provides the following breakdown of groups (Table S3, Supplementary Material). According to the results, a non-significant trend is observed for *Akkermansia muciniphila*, for which the content is lower in the I group compared with the SevI group (*p* = 0.039). *Clostridium perfringens* level was higher in the I group than in the N group (*p* = 0.008) and was numerically higher in the SubI and SevI groups compared with the N group.

We combined the moderate (I) and severe (SevI) insomnia groups to create a single clinical insomnia group (Table S4, Supplementary Material). A higher abundance of *Clostridium perfringens* (*p* = 0.007) was detected in the insomnia group compared with the control. Moreover, the GSTP1 (*p* = 0.034) and *Parvimonas micra* (*p* = 0.046) levels in the insomnia group were numerically lower than in the subclinical insomnia group.

### 3.3. Characteristics of ESS Groups

The Epworth scale questionnaire was used to assess the daytime sleepiness rate. According to the results, the whole sample was divided into two groups (Table S5, Supplementary Material). The content of *Bifidobacterium* spp. (*p* = 0.04), *Eubacterium rectale* (*p* = 0.04), and *Prevotella* spp. (*p* = 0.02) was significantly higher in the excessive daytime sleepiness group compared with the control.

### 3.4. Correlation Analysis Between GSH in Serum and Bacterial Parameters

According to the results, the GSH content in serum is associated with several gut bacteria in the insomnia group only (Table 1).

*S. aureus* was also correlated with GSH in the subclinical insomnia group, but this correlation was stronger, in the insomnia group. Thus, in this group, three species and three genera positively correlate with the GSH content in the serum. However, this tendency disappears in the severe insomnia group. One of the reasons for this could be the size of this group, and hence one transformation was implemented. Combining the two groups (insomnia and severe insomnia) into one yielded the following results: *K. oxytoca*, *S. aureus*, and *M. stadmanae* were still associated with GSH, but only the relationship between *M. stadmanae* and GSH remained strong. Meanwhile, *Prevotella* spp. correlated with this parameter insignificantly. The binomial grouping approach partly confirmed this association.

The analysis of the other questionnaires revealed additional significant bacteria (Table 2).

For the PSQI groups, we only obtained two bacteria with moderate correlations: *S. aureus* and *Shigella* spp. Notably, *S. aureus* did not correlate with GSH in the serum of the control group of either questionnaire with any type of division, but showed moderate to strong correlations in the sleep disorder groups.

Also, *M. stadmanae* exhibited a weak correlation with GSH in the sleep disorder group of PSQI; however, it showed a strong association in the excessive daytime sleepiness group compared with a weak association in the control group.

Furthermore, there was an inverse relationship between GSH in the serum and *E. coli* in the excessive daytime sleepiness group exclusively, as well as between GSH and *Streptococcus* spp.

### 3.5. Correlation Analysis Between GSTP1 in Serum and Bacterial Parameters

Along with GSH in the serum, the GSTP1 concentration was measured. These parameters correlated at a high level (*p* = 2.112 × 10^−13^, rho = 0.69). However, the GSTP1 correlation profile was different (Table 3).

Notably, *S. aureus* again showed significant results. In the insomnia group, *Acinetobacter* spp. was also significantly associated with GSTP1 concentration. In the joint group, *E. rectale* showed a moderate correlation.

On the other hand, in the PSQI groups, the situation was different (Table 4), with an inverse relationship between GSTP1 and *F. prausnitzii* being detected in the control group.

*P. micra* showed a weak correlation with GSTP1 in the ISI SD group, but this correlation was of moderate strength in the excessive daytime sleepiness group.

### 3.6. GSH and GSTP1 Levels in the Presence of Gut Bacteria in Total Group

**Figure 1:**
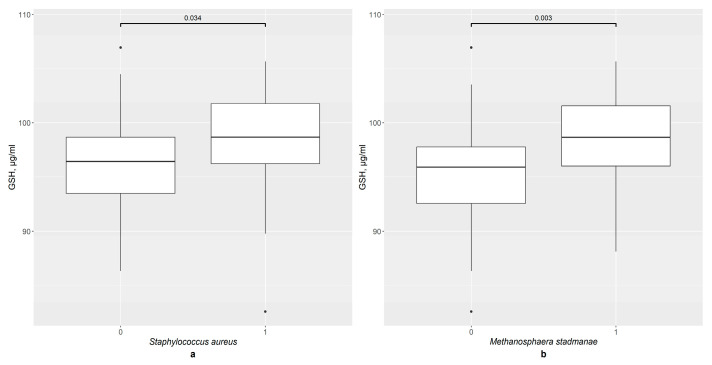
The difference in GSH content in groups: (**a**) 0—*S. aureus* not detected (*n* = 80), 1—*S. aureus* detected (*n* = 16); (**b**) 0—*M. stadmanae* not detected (*n* = 65), 1—*M. stadmanae* detected (*n* = 31).

In the second step of this study, the GSH content in the serum was analyzed in the different groups according to the presence of certain bacteria. In the overall sample, the presence of two bacteria significantly impacted the GSH level: *S. aureus* (Figure 1a), with *p* = 0.034, and *M. stadmanae* (Figure 1b) with *p* = 0.003. Therefore, a more precise analysis was carried out.

### 3.7. GSH and GSTP1 Levels in the Presence of Gut Bacteria in ISI Groups

**Figure 2:**
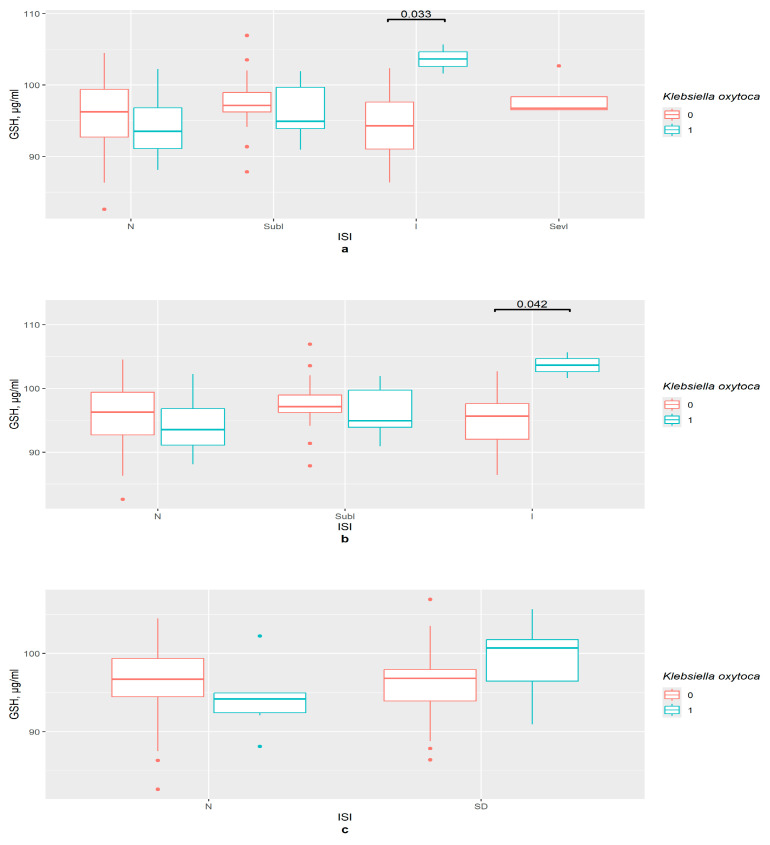
The difference in GSH content in groups (**a**) 0—*K. oxytoca* not detected, 1—*K. oxytoca* detected, N—control (*n*0 = 37, *n*1 = 4), SubI—subclinical insomnia group (*n*0 = 24, *n*1 = 7), I—insomnia group (*n*0 = 17, *n*1 = 2), SevI—severe insomnia group, ISI—insomnia severity index questionnaire; (**b**) 0—*K. oxytoca* not detected, 1—*K. oxytoca* detected, N—control (*n*0 = 37, *n*1 = 4), SubI—subclinical insomnia group (*n*0 = 24, *n*1 = 7), I—combining insomnia group (I and SevI groups) (*n*0 = 21, *n*1 = 3), ISI—insomnia severity index questionnaire; (**c**) 0—*K. oxytoca* not detected, 1—*K. oxytoca* detected, N—control (*n*0 = 46, *n*1 = 6), SD—insomniac sleep disorders group (*n*0 = 36, *n*1 = 8), ISI—insomnia severity index questionnaire.

Firstly, the ISI groups were investigated. *K. oxytoca* influences GSH content in the insomnia group (*p* < 0.05), not in the subclinical insomnia and the control groups (Figure 2a,b). The binomial ISI division provided no statistically significant results (Figure 2c).

**Figure 3:**
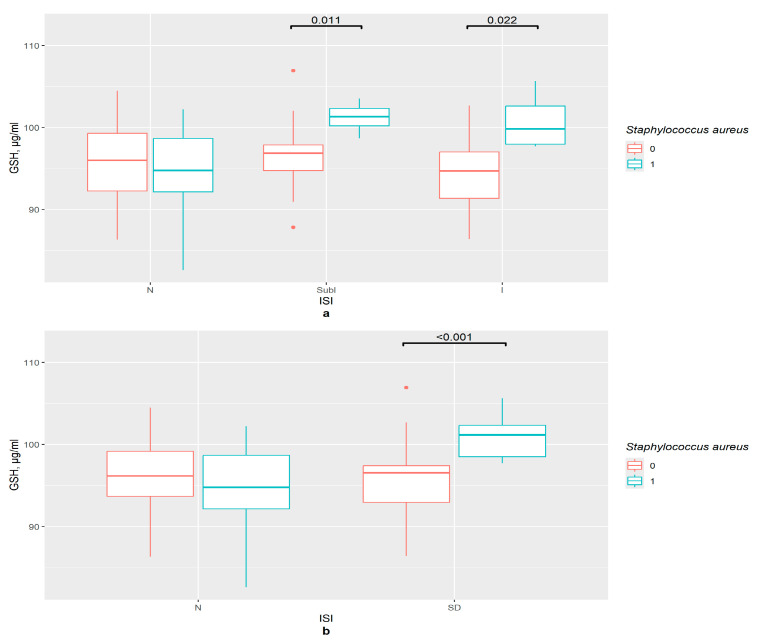
The difference in GSH content in groups (**a**) 0—*S. aureus* not detected, 1—*S. aureus* detected, N—control (*n*0 = 34, *n*0 = 7), SubI—subclinical insomnia group (*n*0 = 27, *n*1 = 4), I—combining insomnia group (I and SevI groups) (*n*0 = 19, *n*1 = 5), ISI—insomnia severity index questionnaire; (**b**) 0—*S. aureus* not detected, 1—*S. aureus* detected, N—control (*n*0 = 45, *n*1 = 7), SD—insomniac sleep disorders group (*n*0 = 35, *n*1 = 9), ISI—insomnia severity index questionnaire.

*S. aureus*’ presence was associated with an increase in the GSH content in the serum of the subclinical (*p* = 0.011) and clinical insomnia groups (*p* = 0.022), but not in that of the control group (Figure 3a). This supports the data obtained in the correlation tests. In the ISI insomnia group with binomial division, GSH concentration was significantly higher in *S. aureus*’ presence (*p* < 0.001), while there was no such tendency for the control group (Figure 3b).

**Figure 4:**
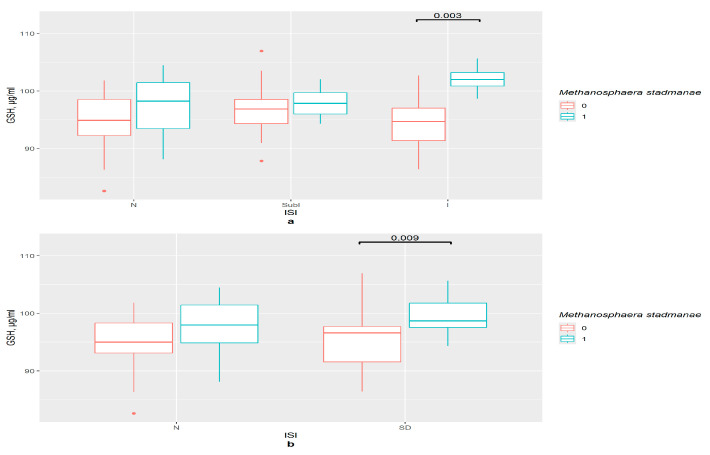
The difference in GSH content in groups (**a**) 0—*M. stadmanae* not detected, 1—*M. stadmanae* detected, N—control (*n*0 = 30, *n*1 = 11), SubI—subclinical insomnia group (*n*0 = 19, *n*1 = 12), I—combining insomnia group (I and SevI groups) (*n*0 = 16, *n*1 = 8), ISI—insomnia severity index questionnaire; (**b**) 0—*M. stadmanae* not detected, 1—*M. stadmanae* detected, N—control (*n*0 = 36, *n*1 = 16), SD—insomniac sleep disorders group (*n*0 = 29, *n*1 = 15), ISI—insomnia severity index questionnaire.

*M. stadmanae* influenced the GSH content in the serum of the insomnia group (*p* = 0.001) and the insomnia and severe insomnia joint group (*p* = 0.003) (Figure 4a). Binomial division presented similar results (*p* = 0.009) (Figure 4b).

**Figure 5:**
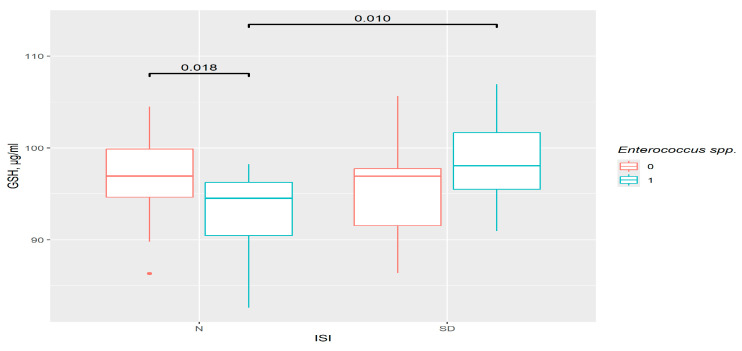
The difference in GSH content in groups. 0—*Enterococcus* spp. not detected, 1—*Enterococcus* spp. detected. N—control (*n*0 = 38, *n*1 = 14), SD—insomniac sleep disorders group (*n*0 = 28, *n*1 = 16), ISI—insomnia severity index questionnaire.

This type of analysis provides one additional result. Insomniac disorders were correlated with a relatively higher level of GSH as compared to the control in the presence of *Enterococcus* spp. (*p* = 0.01). In the control group, the GSH level was significantly lower in presence of this genus (*p* = 0.018) (Figure 5). So, in this case, the difference was not in the insomnia group, but in the control group, suggesting that the participation of bacteria is important.

The concentration of GSTP1 is also different in the presence of *P. micra*, but no associations with sleep disturbances were revealed.

### 3.8. GSH and GSTP1 Levels in the Presence of Gut Bacteria in PSQI Groups

**Figure 6:**
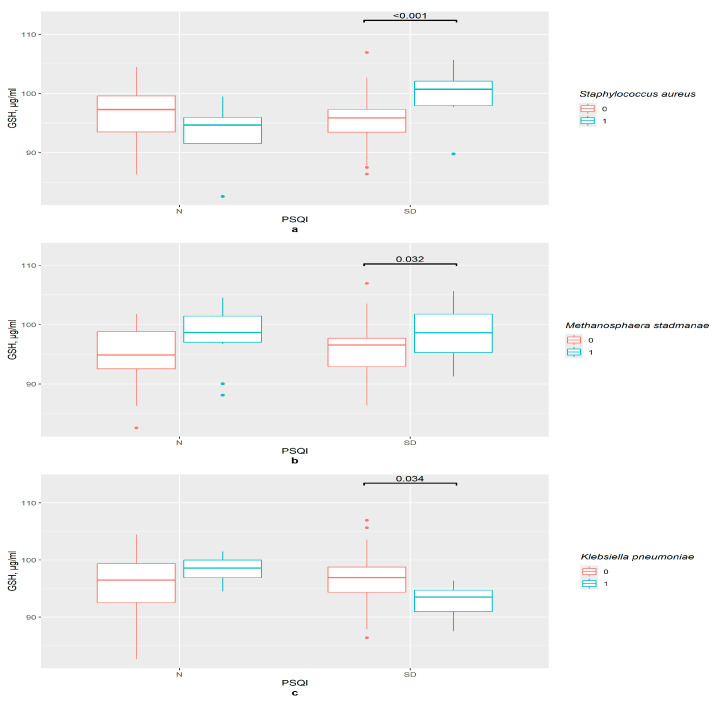
The difference in GSH content in groups: (**a**) 0—*S. aureus* not detected, 1—*S. aureus* detected, N—control (*n*0 = 31, *n*1 = 4), SD—sleep disturbance group (*n*0 = 49, *n*1 = 12), PSQI—Pittsburgh Sleep Quality Index; (**b**) 0—*M. stadmanae* not detected, 1—*M. stadmanae* detected, N—control (*n*0 = 23, *n*1 = 12), SD—sleep disturbance group (*n*0 = 42, *n*1 = 19), PSQI—Pittsburgh Sleep Quality Index; (**c**) 0—*K. pneumoniae* not detected, 1—*K. pneumoniae* detected, N—control (*n*0 = 30, *n*1 = 5), SD—sleep disturbance group (*n*0 = 55, *n*1 = 6), PSQI—Pittsburgh Sleep Quality Index.

In the PSQI control group, there were no important differences in GSH serum concentration between groups divided by the presence or absence of certain bacteria. However, sleep disturbances were associated with variations in the GSH level (*p* = 0.001). The combination of two factors, namely the presence of *S. aureus* and sleep problems, had connections with the increase in GSH content in the serum (Figure 6a).

Noticeably, GSH content was higher in women with sleep disturbances in the presence of *M. stadmanae* compared with the group with an absence of this bacteria (*p* = 0.032); however, no significant differences between PSQI groups were observed (Figure 6b).

So, for these two bacteria, sleep disturbances were an important factor for GSH content level. Two other bacteria provided no significant results when other methods of dividing the groups were applied. In the presence of *K. pneumoniae*, the GSH level is lower in the sleep disturbances group (*p* = 0.034) (Figure 6c).

**Figure 7:**
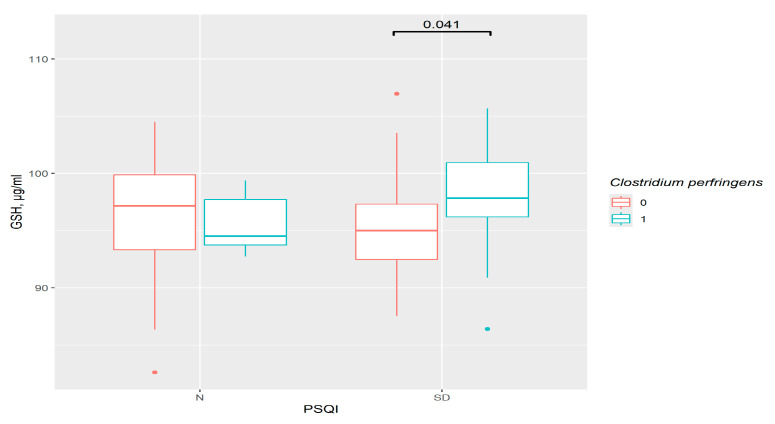
The difference in GSH content in groups: 0—*C. perfringens* not detected, 1—*C. perfringens* detected, N—control (*n*0 = 30, *n*1 = 5), SD—sleep disturbance group (*n*0 = 39, *n*1 = 22), PSQI—Pittsburgh Sleep Quality Index.

A higher level of GSH was observed in the *C. perfringens* group with sleep disorders (*p* = 0.041) (Figure 7). However, the variations between PSQI groups in the presence or absence of this bacteria were invalid.

GSTP1 content fluctuations were non-significant.

### 3.9. GSH and GSTP1 Levels in the Presence of Gut Bacteria in ESS Groups

**Figure 8:**
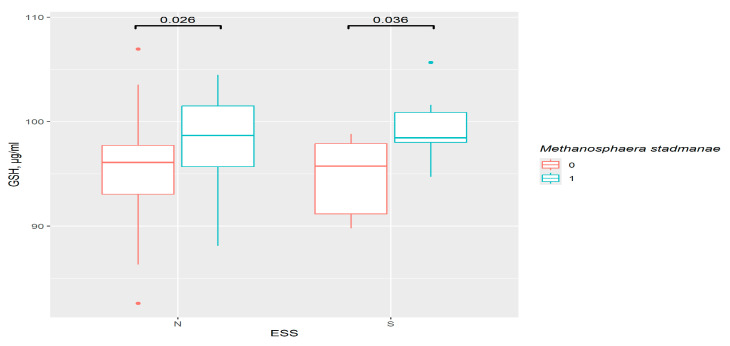
The difference in GSH content in groups: 0—*M. stadmanae* not detected, 1—*M. stadmanae* detected, N—control (*n*0 = 56, *n*1 = 23), S—excessive daytime sleepiness (*n*0 = 9, *n*1 = 8), ESS—Epworth Sleepiness Scale.

In ESS groups, the only important result was the increase in GSH in presence of *M. stadmanae* (*p* = 0.026 in the control and *p* = 0.036 in the excessive daytime sleepiness group). However, this was related to the bacteria, rather than sleep disorders, as observed for the PSQI (Figure 8).

**Figure 9:**
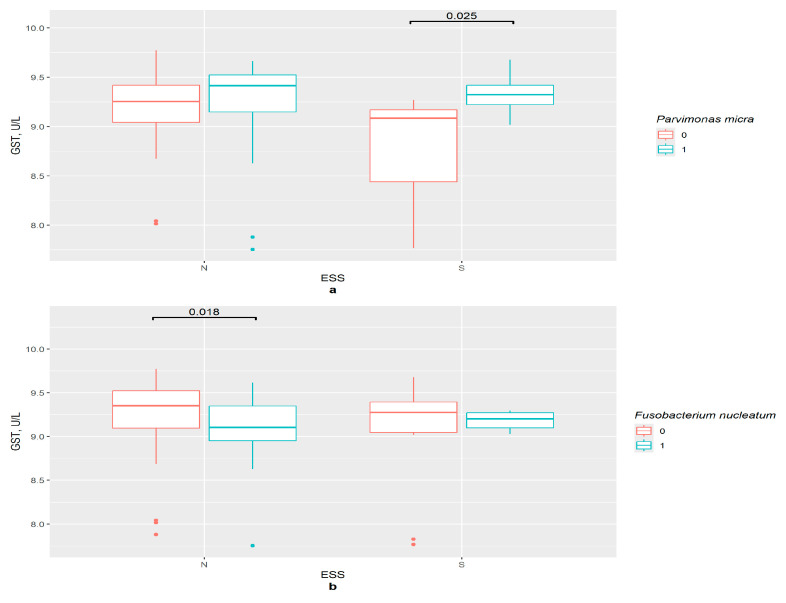
The difference in GSTP1 content in groups:: (**a**) 0—*P. micra* not detected, 1—*P. micra* detected, N—control (*n*0 = 49, *n*1 = 30), S—excessive daytime sleepiness (*n*0 = 7, *n*1 = 10), ESS—Epworth Sleepiness Scale; (**b**) 0—*F. nucleatum* not detected, 1—*F. nucleatum* detected, N—control (*n*0 = 60, *n*1 = 19), S—excessive daytime sleepiness (*n*0 = 12, *n*1 = 5), ESS—Epworth Sleepiness Scale.

In the absence of *P. micra*, the GSTP1 concentration was lower in the excessive daytime sleepiness group compared with the control (*p* = 0.025) (Figure 9a). In the control group, the GSTP1 content was lower in the presence of *F. nucleatum* (*p* = 0.018). However, no differences between ESS groups in the *F. nucleatum* absence/presence subgroups were observed (Figure 9b).

### 3.10. GSH and GSTP1 Association with Bacteria in Different Groups with Sleep Disorders

Many species and genera are therefore associated with glutathione system fluctuations. GSH in the serum was mostly higher in cases of sleep disturbances and in the presence of certain bacteria. Moreover, it was correlated with bacteria content in the gut (Table 5).

GSH level in the serum correlated with GSTP1 concentration; however, the GSTP1–microbiome correlation profile was different (Table 6).

## 4. Discussion

In recent years, studies of the human microbiome have led to development of the gut–brain axis theory, which proposes a relationship between the gut bacterial community, along with its metabolites and chemical compounds, and physiological and pathological processes in the brain. The number of studies that have addressed the link between the menopause, the gut microbiome, and sleep is relatively small. Moreover, sleep is not a standalone characteristic, and is rather considered as part of a set of other factors [29]. It must be mentioned that the profiles of people with insomniac disorder investigated in other studies do differ. Some authors have suggested that *Prevotella* plays an important role in insomnia, potentially affecting sleep by regulating amino acid metabolism and promoting inflammation [13], because *Prevotella* correlates with serum indoxyl sulfate levels [30]. Indoxyl sulfate, a tryptophan derivative, is produced by intestinal bacteria. It is a uremic toxin, promoting inflammation and oxidative stress [31]. In our study, the level of *Prevotella* was found to be higher in the group with excessive daytime sleepiness and was associated with GSH only in the insomnia group, and not in the control or subclinical insomnia group. Therefore, it is possible that under sleep deficiency conditions, this bacteria may be associated with oxidative stress development.

Another study revealed that *Streptococcus*’ relative abundance was considerably higher in insomniacs compared to healthy controls [32]. Our results reveal only a negative correlation between *Streptococcus* and GSH in the group with the excessive daytime sleepiness. There are other controversial reports regarding *Streptococcus* trends in patients with insomnia disorder [33]. Thus, more precise investigations are needed to evaluate these results.

The investigation of the correlation between gut content and serum metabolites revealed that the levels of the *Bacteroidaceae* and *Ruminococcaceae* families significantly decreased in patients with insomnia disorder compared with healthy controls. On the other hand, levels of the *Prevotellaceae* family increased. At the genus level, a significant decrease in levels of the genus *Bacteroides* and a significant increase in levels of the genus *Prevotella* were observed for patients with insomnia disorder, compared with healthy controls [34].

In our study, *Faecalibacterium prausnitzii* showed a negative correlation with GSTP1 in the PSQI control group and a positive correlation in the ISI insomniac group. It is claimed that the increased resistance of the bacteria correlates with the presence of oxygen superoxide or ROS-detoxifying enzymes and genes [35]. Oxidative stress could be an unfavorable condition for this genus, so in the control, the decreased GSTP1 concentration might mean a more preferable condition for this bacteria; meanwhile, in the sleep deficiency group, the increased GSTP1 level may be aimed at reducing oxidative stress.

In an animal model, the relative abundances of *Escherichia*–*Shigella* increased in the induced insomnia group [36]. An association between *Escherichia*–*Shigella* content and dietary habits in insomnia was also detected [37]. According to our results, *E. coli* negatively correlates with GSH only in the excessive daytime sleepiness group. However, *Shigella* spp. demonstrates a moderate association with GSH in the insomniac and sleep disorder groups for all questionnaires. It is notable that two bacteria species display similar results—*S. aureus* and *M. stadmanae* apparently have a strong association with GSH content in sleep deficiency terms. Unlike *Shigella* spp., these significant results were detected not only in quantitative but also in qualitative analysis of this bacteria’s abundance. *S. aureus* is related to GSTP1 content in the insomnia group as well.

We reported previously that *M. stadmanae* methanogen is associated with a higher level of TBARS in the group with sleep disorders. Moreover, TBARS and advanced oxidation protein products increase in the group with sleep disorders compared to the control in the sample with *M. stadtmanae* in the gut [20]. Therefore, the presented parameters appear promising for further investigation.

*E. coli* and *Klebsiella* overgrowth is assumed as the pathophysiological mechanism behind gut dysbiosis [38]. *K. oxytoca* positively correlates with GSH content in the serum in insomnia groups, and GSH level was higher in the presence of this bacteria. On the other hand, *K. pneumoniae*’s presence is associated with a lower GSH level in the SD group of PSQI. The indole production ability is the main difference between these two bacteria [39]. Indole derivatives influence GSTP1 concentration [40]. Despite a direct impact on GSTP1 not being observed in our study, this mechanism could explain the difference in GSH results. It is well known that indole derivatives mitigate oxidative stress and inflammation [41,42]. Therefore, it is possible that the glutathione system is one of the main ways for this activity to occur.

It is possible that oxidative stress or sleep alterations may be a reason for or a consequence of microbial fluctuations. GSH supplementation influences gut microbiome diversity. The abundance of the phylum *Proteobacteria* significantly decreased, as well as that of pathogenic *Escherichia*/*Shigella*; however, genera such as *Megasphaera*, *Bacteroides*, and *Megamonas* were found to be significantly enriched after supplementation [43]. On the other hand, modeling postmenopausal conditions in mice showed that Firmicutes and Bacteroidetes regulate the key GSH enzyme glutamate–cysteine ligase catalytic subunit and inhibit mitochondrial biogenesis and reactive oxygen species accumulation via the cAMP response element-binding (CREB) pathway. This affects the de novo synthesis of GSH [44]. Recent studies have shown that the gut microbiota is interconnected with circadian rhythms. Short-chain fatty acids or bile acids produced by the gut microbiota may mediate this relationship. The influence of microbiota metabolites on circadian rhythms is extensive and is linked to their other functions, such as their participation in energy metabolism and immune responses [45,46].

Menopause-related hormone changes may also affect the gut microbiome composition. It has been suggested that the depletion of ovarian steroid hormones has an impact on the deconjugation of glucuronide or sulfate groups from sex steroid hormones, allowing for enterohepatic recirculation. This is supported by studies which revealed differences in the gut content of individuals of different genders. Additionally, changes were also detected after puberty. On the other hand, the complex effects of the menopause have been investigated insufficiently [47,48,49,50,51,52,53,54], with information about the state of the glutathione system in menopausal women with insomnia clearly lacking. According to previous results, the menopausal status is clearly relevant for the state of the glutathione system [21].

One potential mechanism linking the gut microbiota and sleep disorders is the hormone melatonin [55], which effectively decreases oxidative stress markers and increases the levels of antioxidative factors, including GSH [56,57]. On the other hand, being produced in the gastrointestinal tract and having local and systemic effects, melatonin is closely connected to the intestinal microbiota [58]. Melatonin alters the composition of the gut microbiota, decreasing or increasing the levels of certain genera, restoring disturbances caused by sleep problems, stress, or other negative factors. The mechanism of this effect is debated, but it is believed that butyrate plays an important role in mediating it. It is also suggested that the composition of the microbiome influences melatonin levels [55]. There is much evidence of the important role of melatonin in the association between oxidative stress and the gut microbiome, but it should be mentioned that most studies in this field are based on animal models, with a lack of human investigations [9,59]. It is possible that the further research in this field may provide more significant information about the discussed processes.

There are limitations to this study, the first of which is the small sample size employed. However, to justify the obtained results, the groups were combined, and additional methods of dividing the sample were applied. Meanwhile, taxa with insufficient registered results were tested as quality factors. Therefore, the findings which were not registered in multiple tests should be interpreted cautiously. Secondly, the disproportionate distribution of women across groups could cause difficulties in result assessment. Further investigations are therefore needed. Additionally, the fact that the questionnaires were self-administered might lead to misunderstandings and further mistakes with the grouping of volunteers. However, we provided consultation to minimize unfavorable consequences. Lastly, the sleep questionnaires used have not been not validated for our region. On the other hand, many studies have proven their reliability.

## 5. Conclusions

The association between certain gut bacteria in menopausal women and serum oxidative stress markers was revealed, with the key role of sleep disturbances in this association being determined. It is possible that the correlations between the gut microbiome and oxidative stress in menopausal women with sleep disturbances extend beyond the glutathione system in the serum, and further research may focus on other parameters affecting this relationship.

## Figures and Tables

**Table 1:** Spearman correlation between GSH in serum and bacterial parameters in ISI groups.

Parameter 1	ISI N	ISI SubI	rho CI	ISI I	rho CI	ISI SevI	ISI2 N	ISI2 SD	rho CI	ISI I+SevI	rho CI
*E. coli*	0.14	0.79		0.46		0.33	0.31	0.59		0.48	
*K. oxytoca*	0.61	0.54		0.04	0.53 [0.31; 0.76]	NA	0.27	0.11		0.04	0.46 [0.22; 0.69]
*S. aureus*	0.75	0.01	0.45 [0.24; 0.68]	0.01	0.60 [0.25; 0.86]	NA	0.67	0.00	0.52 [0.32; 0.70]	0.02	0.53 [0.23; 0.79]
*Enterobacter* spp.	0.21	0.56		0.01	0.61 [0.10; 0.84]	0.74	0.22	0.02	0.36 [0.06; 0.60]	0.05	
*Shigella* spp.	0.11	0.07		0.04	0.53 [0.31; 0.76]	NA	0.11	0.00	0.44 [0.25; 0.63]	0.04	0.46 [0.22; 0.69]
*Streptococcus* spp.	0.66	0.82		0.06		0.42	0.80	0.11		0.10	
*Prevotella* spp.	0.54	0.99		0.01	0.62 [0.30; 0.84]	0.26	0.56	0.14		0.05	
*M. stadmanae*	0.21	0.19		0.00	0.76 [0.42; 0.91]	NA	0.07	0.01	0.43 [0.15; 0.65]	0.00	0.64 [0.37; 0.89]

NA—not available (too little data for the test), Spearman’s rank correlation coefficient (rho) was provided only for statistically significant results, CI—confidence intervals.

**Table 2:** Spearman correlation between GSH in serum and bacterial parameters in groups.

Parameter 1	Total	rho CI	PSQI N	PSQI SD	rho CI	ESS N	rho CI	ESS S	rho CI
*E*. *coli*	0.21		0.72	0.07		0.63		0.04	−0.52 [−0.85; 0.08]
*K*. *oxytoca*	0.75		0.07	0.23		0.86		0.34	
*S*. *aureus*	0.04	0.22 [−0.03; 0.41]	0.34	0.00	0.43 [0.12; 0.61]	0.14		0.29	
*Enterobacter* spp.	0.75		0.56	0.33		0.85		0.39	
*Shigella* spp.	0.00	0.34 [0.21; 0.49]	NA	0.00	0.44 [0.28. 0.61]	0.02	0.27 [0.15; 0.45]	0.02	0.59 [0.43; 0.75]
*Streptococcus* spp.	0.20		0.80	0.20		0.88		0.01	−0.67 [−0.90; −0.14]
*Prevotella* spp.	0.64		0.70	0.29		0.82		0.18	
*M*. *stadmanae*	0.00	0.33 [0.12; 0.51]	0.08	0.02	0.31 [0.06; 0.54]	0.02	0.27 [0.03; 0.47]	0.01	0.63 [0.18; 0.86]

NA—not available (too little data for the test), Spearman’s rank correlation coefficient (rho) was provided only for statistically significant results, CI—confidence intervals.

**Table 3:** Spearman correlation between GSTP1 and bacterial parameters in ISI groups.

Parameter 1	ISIsc N	ISIsc SubI	rho CI	ISIsc I	rho CI	ISIsc SevI	ISI2 N	ISI2 SD	rho CI	ISI3 I	rho CI
*F. prausnitzii*	0.11	0.26		0.23		0.35	0.10	0.83		0.09	
*S. aureus*	0.25	0.01	0.45 [0.27; 0.70]	0.05		0.18	0.17	0.09		0.37	
*P. micra*	0.80	0.22		0.34		0.18	0.69	0.03	0.32 [0.03; 0.56]	0.76	
*Acinetobacter* spp.	0.92	0.84		0.03	0.50 [0.11; 0.75]	0.17	0.68	0.18		0.19	
*E. rectale*	0.06	0.47		0.07		0.55	0.07	0.63		0.04	0.42 [−0.04; 0.71]

Spearman’s rank correlation coefficient (rho) was provided only for statistically significant results.

**Table 4:** Spearman correlation between GSTP1 and bacterial parameters in groups.

Parameter 1	Total	PSQI N	rho CI	PSQI SD	ESS N	ESS S	rho CI
*F. prausnitzii*	0.39	0.01	−0.45 [−0.67; −0.14]	0.61	0.57	0.63	
*S. aureus*	0.63	0.16		0.15	0.69	0.66	
*P. micra*	0.07	0.46		0.13	0.22	0.05	0.49 [0.06; 0.77]
*Acinetobacter* spp.	0.21	0.63		0.09	0.21	0.59	
*E. rectale*	0.37	0.10		0.80	0.48	0.52	

Spearman’s rank correlation coefficient (rho) was provided only for statistically significant results.

**Table 5:** GSH Association with Bacteria in Different Groups with Sleep Disorders.

Parameter	PSQI	ISI 4 Groups	ISI 3 Groups	ISI 2 Groups	ESS
*E. coli*					Negative, moderate in S group
*K. oxytoca*		Positive, moderate in Insomnia group	Positive, moderate in combining group		
*S. aureus*	Positive, moderate in SD group	Positive, moderate in SubI group; positive, strong in I group	Positive, moderate in combining group	Positive, moderate in I group	
*Enterobacter* spp.		Positive, strong in I group		Positive, weak in I group	
*Shigella* spp.	Positive, moderate in SD group	Positive, moderate in Insomnia group	Positive, moderate in combining group	Positive, moderate in I group	Positive, weak in control; positive, moderate in S group
*Streptococcus* spp.					Negative, strong in S group
*Prevotella* spp.		Positive, strong in I group			
*M. stadmanae*	Positive, moderate in SD group	Positive, strong in I group	Positive, strong in combining group	Positive, moderate in I group	Positive, weak in control; positive, strong in S group
*K. oxytoca* bi		Higher in 1 than in 0 in I group	Higher in 1 than in 0 in combining group		
*S. aureus* bi	Higher in 1 than in 0 in SD group	Higher in 1 than in 0 in I and SubI groups	Higher in 1 than in 0 in combining and SubI groups	Higher in 1 than in 0 in I group	
*Enterococcus* spp. bi				Higher in I than in control in 1 group	
*K. pneumoniae* bi	Lower in SD than in control in 1 group				
*M. stadmanae* bi		Higher in 1 than in 0 in I group	Higher in 1 than in 0 in combining group	Higher in 1 than in 0 in I group	

**Table 6:** GSTP1 association with bacteria in different groups with sleep disorders.

Parameter	PSQI	ISI 4 Groups	ISI 3 Groups	ISI 2 Groups	ESS
*F. prausnitzii*	Negative, moderate in Control			Positive, moderate in I group	
*S. aureus*		Positive, moderate in SubI group; positive, moderate in I group			
*P. micra*				Positive, weak in I group	Positive, moderate in S group
*Acinetobacter* spp.		Positive, moderate in I group			
*E. rectale*			Positive, moderate in combining group		
*F. nucleatum* bi					Lower in 1 than 0 in Control
*P. micra* bi					Lower in S than in control in 0

## Data Availability

The original contributions presented in this study are included in the article/Supplementary Materials. Further inquiries can be directed to the corresponding author.

## Supplementary Materials

The following supporting information can be downloaded at: https://www.mdpi.com/article/10.3390/pathophysiology33010003/s1, such as characteristics of PSQI groups and the total sample; characteristics of ISI groups, binomial division; characteristics of ISI groups, standard breakdown; characteristics of ISI groups, combining insomniac disorders in one group; characteristics of ESS groups.
