# Supplementary material for: Sleep Disorders in Climacteric Women: Glutathione, Glutathione S-Transferase P1 and Gut Microbiome Interrelation
Source: Pathophysiology. 2025 Dec 26;33(1):3. doi: 10.3390/pathophysiology33010003 (PMC12821644; doi:10.3390/pathophysiology33010003)
Supplement: Supplementary file 1 [file pathophysiology-33-00003-s001.zip › pathophysiology-4037762-supplementary.pdf]

Supplementary material

Table S1. Characteristics of PSQI groups and the total sample

	**N (N=35)**	**SD (N=61)**	**p-value (Wilcoxon)**	**Overall (N=96)**
**Age**	58.0 [54.0; 63.5]	56.0 [51.0; 62.0]	0.15	57.0 [51.0; 62.3]
**BMI**	25.6 [23.3; 28.4]	26.8 [23.9; 29.1]	0.14	26.3 [23.7; 28.8]
**Total bacterial mass**	13.0 [12.7; 13.3]	13.0 [12.7; 13.3]	0.98	13.0 [12.7; 13.3]
***Lactobacillus* spp.**	6.90 [6.48; 7.60]	6.85 [6.48; 7.70]	0.65	6.87 [6.48; 7.63]
***Bifidobacterium* spp.**	11.0 [10.3; 11.6]	11.0 [10.3; 11.8]	0.49	11.0 [10.3; 11.7]
Escherichia coli	8.90 [8.30; 9.54]	8.95 [8.30; 9.78]	0.76	8.95 [8.30; 9.72]
***Bacteroides* spp.**	12.8 [12.2; 13.2]	12.7 [12.5; 13.0]	0.85	12.7 [12.5; 13.1]
Faecalibacterium prausnitzii	11.8 [11.7; 12.2]	12.0 [11.8; 12.3]	0.13	12.0 [11.7; 12.3]
Bacteroides thetaomicron	8.60 [0; 9.81]	8.30 [0; 9.60]	0.75	8.48 [0; 9.78]
Akkermansia muciniphila	9.95 [8.15; 10.9]	9.60 [5.70; 11.3]	0.88	9.65 [6.79; 11.3]
***Enterococcus* spp.**	**0 [0; 0]**	**0 [0; 5.90]**	**0.03**	0 [0; 5.48]
***Escherichia coli* enteropathogenic**	0 [0; 0]	0 [0; 0]	0.46	0 [0; 0]
Klebsiella pneumoniae	0 [0; 0]	0 [0; 0]	0.60	0 [0; 0]
Klebsiella oxytoca	0 [0; 0]	0 [0; 0]	0.07	0 [0; 0]
***Candida* spp.**	0 [0; 0]	0 [0; 0]	0.77	0 [0; 0]
Staphylococcus aureus	0 [0; 0]	0 [0; 0]	0.33	0 [0; 0]
Clostridium difficile	0 [0; 0]	0 [0; 0]	-	0 [0; 0]
Clostridium perfringens	**0 [0; 0]**	**0 [0; 6.70]**	**0.01**	0 [0; 5.70]
Proteus vulgaris mirabilis	0 [0; 0]	0 [0; 0]	0.65	0 [0; 0]
***Citrobacter* spp.**	0 [0; 0]	0 [0; 0]	0.20	0 [0; 0]
***Enterobacter* spp.**	0 [0; 7.30]	0 [0; 7.70]	0.88	0 [0; 7.40]
Fusobacterium nucleatum	0 [0; 0]	0 [0; 5.00]	0.73	0 [0; 1.25]
Parvimonas micra	0 [0; 6.84]	0 [0; 6.00]	0.40	0 [0; 6.48]

	N (N=35)	SD (N=61)	p-value (Wilcoxon)	Overall (N=96)
Salmonella **spp.**	0 [0; 0]	0 [0; 0]	-	0 [0; 0]
Shigella **spp.**	**0 [0; 0]**	**0 [0; 0]**	**0.04**	0 [0; 0]
Blautia **spp.**	8.48 [0; 9.54]	8.30 [0; 9.70]	0.99	8.39 [0; 9.70]
Acinetobacter **spp.**	7.30 [7.00; 7.60]	7.30 [6.78; 7.70]	0.66	7.30 [6.95; 7.70]
Streptococcus **spp.**	7.78 [6.77; 8.81]	7.60 [6.00; 8.60]	0.20	7.60 [6.60; 8.70]
Eubacterium rectale	10.8 [10.2; 11.4]	11.0 [10.3; 11.5]	0.63	10.9 [10.3; 11.5]
Roseburia inulinivorans	10.6 [10.0; 11.3]	10.8 [9.95; 11.3]	0.61	10.8 [10.0; 11.3]
Prevotella **spp.**	9.00 [6.22; 11.2]	10.0 [7.48; 12.0]	0.22	9.48 [7.48; 11.6]
Methanobrevibacter smithii	8.30 [0; 9.00]	0 [0; 8.30]	0.17	3.30 [0; 8.95]
Methanosphaera stadmanae	0 [0; 6.65]	0 [0; 5.48]	0.76	0 [0; 5.93]
Ruminococcus **spp.**	7.85 [5.93; 9.00]	7.70 [6.48; 8.95]	0.81	7.81 [6.48; 9.00]
**GSTP1**	9.35 [9.08; 9.50]	9.27 [9.03; 9.43]	0.33	9.27 [9.08; 9.47]
**GSH**	96.9 [93.5; 99.4]	96.7 [94.0; 98.7]	0.84	96.7 [93.9; 99.1]

N is a control group with PSQI score less than 5; SD is a group with sleep disturbances, PSQI score more than 5. Between-group differences are shown in bold ($p < 0.05$).

Table S2. Characteristics of ISI groups, binomial division

	N (N=52)	SD (N=44)	p-value (Wilcoxon)
**Age**	58.0 [53.5; 64.0]	55.5 [50.8; 61.3]	0.07
**BMI**	26.2 [23.7; 28.7]	27.1 [23.7; 29.1]	0.64
**Total bacterial mass**	**13.0 [12.8; 13.5]**	**12.8 [12.6; 13.3]**	**0.03**
Lactobacillus **spp.**	7.00 [6.48; 7.70]	6.77 [6.30; 7.35]	0.24
Bifidobacterium **spp.**	11.0 [10.3; 11.6]	11.0 [10.3; 11.9]	0.54
Escherichia coli	8.98 [8.23; 9.86]	8.93 [8.43; 9.38]	0.96
Bacteroides **spp.**	12.8 [12.5; 13.3]	12.6 [12.4; 12.9]	0.08
Faecalibacterium prausnitzii	12.0 [11.7; 12.3]	12.0 [11.7; 12.3]	0.74

	N (N=52)	SD (N=44)	p-value (Wilcoxon)
Bacteroides thetaomicron	9.15 [0; 10.3]	8.00 [0; 9.30]	0.13
Akkermansia muciniphila	9.80 [7.50; 11.0]	9.54 [6.64; 11.3]	0.93
***Enterococcus* spp.**	0 [0; 5.12]	0 [0; 5.58]	0.34
***Escherichia coli* enteropathogenic**	0 [0; 0]	0 [0; 0]	0.29
Klebsiella pneumoniae	0 [0; 0]	0 [0; 0]	0.06
Klebsiella oxytoca	0 [0; 0]	0 [0; 0]	0.36
***Candida* spp.**	0 [0; 0]	0 [0; 0]	0.08
Staphylococcus aureus	0 [0; 0]	0 [0; 0]	0.37
Clostridium difficile	0 [0; 0]	0 [0; 0]	-
Clostridium perfringens	**0 [0; 0]**	**0 [0; 6.97]**	**0.002**
Proteus vulgaris mirabilis	0 [0; 0]	0 [0; 0]	0.75
***Citrobacter* spp.**	0 [0; 0]	0 [0; 0]	0.22
***Enterobacter* spp.**	0 [0; 8.51]	0 [0; 7.00]	0.40
Fusobacterium nucleatum	0 [0; 5.35]	0 [0; 0]	0.31
Parvimonas micra	0 [0; 6.78]	0 [0; 5.97]	0.64
***Salmonella* spp.**	0 [0; 0]	0 [0; 0]	-
***Shigella* spp.**	**0 [0; 0]**	**0 [0; 0]**	**0.03**
***Blautia* spp.**	8.39 [0; 9.51]	8.24 [0; 9.72]	0.82
***Acinetobacter* spp.**	7.30 [7.00; 7.78]	7.30 [6.36; 7.60]	0.34
***Streptococcus* spp.**	**7.84 [6.89; 8.87]**	**7.30 [6.00; 8.48]**	**0.03**
Eubacterium rectale	11.0 [10.4; 11.5]	10.7 [10.2; 11.3]	0.30
Roseburia inulinivorans	10.9 [10.2; 11.3]	10.7 [9.53; 11.3]	0.24
***Prevotella* spp.**	9.48 [7.19; 11.5]	9.80 [7.48; 11.9]	0.97
Methanobrevibacter smithii	7.10 [0; 9.00]	0 [0; 8.30]	0.35
Methanosphaera stadmanae	0 [0; 5.58]	0 [0; 6.08]	0.73
***Ruminococcus* spp.**	7.85 [5.94; 8.97]	7.70 [6.64; 9.00]	0.98
**GSTP1**	9.27 [9.08; 9.46]	9.28 [9.05; 9.50]	0.94
**GSH**	96.1 [93.6; 99.2]	96.9 [94.1; 98.7]	0.58

N is a control group, SD – a group with insomniac sleep disturbances. Between-group differences are shown in bold ($p < 0.05$).

Table S3. Characteristics of ISI groups, standard breakdown; Kruskal-Wallis p-value

	N (N=41)	SubI (N=31)	I (N=19)	SevI (N=5)	p-value
**Age**	58.0 [54.0; 64.0]	56.0 [50.5; 61.5]	56.0 [51.0; 61.0]	50.0 [50.0; 58.0]	0.20
**BMI**	26.6 [24.8; 30.7]	24.7 [23.4; 27.2]	27.7 [23.8; 30.2]	28.1 [27.8; 30.8]	0.09
**Total bacterial mass**	13.0 [12.8; 13.5]	12.9 [12.7; 13.3]	12.8 [12.5; 13.2]	13.0 [12.8; 13.3]	0.29
***Lactobacillus* spp.**	6.70 [6.48; 7.60]	7.00 [6.54; 7.92]	6.70 [6.00; 7.24]	6.70 [6.48; 6.90]	0.29
***Bifidobacterium* spp.**	11.0 [10.3; 11.6]	10.9 [10.1; 11.8]	11.0 [10.7; 11.5]	11.0 [10.8; 11.9]	0.97
Escherichia coli	8.95 [8.00; 9.70]	9.30 [8.54; 9.84]	8.78 [7.80; 9.00]	8.30 [8.00; 10.3]	0.29
***Bacteroides* spp.**	12.8 [12.5; 13.3]	12.7 [12.4; 13.2]	12.6 [12.4; 13.0]	12.8 [12.7; 13.0]	0.61
Faecalibacterium prausnitzii	11.9 [11.7; 12.3]	12.0 [11.7; 12.4]	12.0 [11.8; 12.3]	12.0 [11.3; 12.3]	0.90
Bacteroides thetaomicron	9.30 [0; 10.3]	7.90 [0; 9.45]	8.30 [6.48; 9.15]	8.30 [6.30; 9.30]	0.61
Akkermansia muciniphila	9.90 [8.48; 11.0]	10.3 [7.30; 11.4]	7.00 [0; 9.69]	11.5 [11.5; 11.5]	0.02
***Enterococcus* spp.**	0 [0; 5.00]	0 [0; 6.00]	0 [0; 2.74]	0 [0; 0]	0.38
***Escherichia coli* enteropathogenic**	0 [0; 0]	0 [0; 0]	0 [0; 0]	0 [0; 0]	0.55
Klebsiella pneumoniae	0 [0; 0]	0 [0; 0]	0 [0; 0]	0 [0; 0]	0.24
Klebsiella oxytoca	0 [0; 0]	0 [0; 0]	0 [0; 0]	0 [0; 0]	0.45
***Candida* spp.**	0 [0; 0]	0 [0; 0]	0 [0; 0]	0 [0; 0]	0.84

	N (N=41)	SubI (N=31)	I (N=19)	SevI (N=5)	p-value
Staphylococcus aureus	0 [0; 0]	0 [0; 0]	0 [0; 0]	0 [0; 0]	0.85
Clostridium difficile	0 [0; 0]	0 [0; 0]	0 [0; 0]	0 [0; 0]	-
Clostridium perfringens	**0 [0; 0]**	0 [0; 6.13]	**5.30 [0; 7.15]**	0 [0; 5.70]	0.02
Proteus vulgaris mirabilis	0 [0; 0]	0 [0; 0]	0 [0; 0]	0 [0; 0]	0.74
Citrobacter spp.	0 [0; 0]	0 [0; 0]	0 [0; 0]	0 [0; 0]	0.50
Enterobacter spp.	0 [0; 7.70]	5.60 [0; 8.50]	0 [0; 6.30]	0 [0; 5.85]	0.69
Fusobacterium nucleatum	0 [0; 5.60]	0 [0; 0]	0 [0; 5.00]	0 [0; 0]	0.31
Parvimonas micra	0 [0; 6.48]	5.70 [0; 6.78]	0 [0; 0]	0 [0; 0]	0.11
Salmonella spp.	0 [0; 0]	0 [0; 0]	0 [0; 0]	0 [0; 0]	-
Shigella spp.	0 [0; 0]	0 [0; 0]	0 [0; 0]	0 [0; 0]	0.34
Blautia spp.	8.30 [0; 9.30]	8.48 [0; 9.74]	7.60 [0; 9.59]	8.48 [7.30; 10.3]	0.83
Acinetobacter spp.	7.30 [7.00; 7.60]	7.30 [7.00; 7.81]	7.30 [6.63; 7.60]	7.00 [0; 7.30]	0.58
Streptococcus spp.	7.78 [6.70; 8.95]	7.48 [6.84; 8.50]	6.60 [5.95; 8.48]	7.60 [6.48; 8.48]	0.22
Eubacterium rectale	11.0 [10.5; 11.5]	10.8 [10.1; 11.5]	10.7 [10.4; 11.3]	11.0 [10.7; 11.0]	0.81
Roseburia inulinivorans	10.9 [10.3; 11.5]	10.8 [9.69; 11.3]	10.9 [9.92; 11.3]	10.5 [8.85; 10.7]	0.37
Prevotella spp.	9.30 [6.60; 11.3]	9.48 [8.15; 12.0]	10.5 [9.48; 12.0]	7.48 [7.48; 8.78]	0.28

	N (N=41)	SubI (N=31)	I (N=19)	SevI (N=5)	p-value
Methanobrevibacter smithii	0 [0; 8.95]	0 [0; 9.00]	6.60 [0; 8.00]	7.90 [0; 8.30]	0.96
Methanosphaera stadmanae	0 [0; 5.30]	0 [0; 6.15]	0 [0; 7.13]	0 [0; 0]	0.65
Ruminococcus spp.	8.00 [7.00; 9.00]	6.90 [2.85; 8.00]	8.00 [7.15; 8.92]	7.85 [7.70; 9.00]	0.08
GSTP1	9.25 [9.06; 9.47]	9.36 [9.21; 9.52]	9.20 [8.95; 9.33]	9.24 [9.09; 9.30]	0.10
GSH	95.7 [92.1; 99.4]	97.0 [94.9; 99.0]	94.7 [91.4; 98.2]	96.8 [96.6; 98.4]	0.44

N is a control group; SubI – subclinical insomnia group; I – clinical insomnia group; SevI – clinical severe insomnia group. Between-group differences are shown in bold (p < 0.013).

Table S4. Characteristics of ISI groups, combining insomniac disorders in one group; Kruskal-Wallis p-value

	N (N=41)	SubI (N=31)	I (N=24)	p-value
**Age**	58.0 [54.0; 64.0]	56.0 [50.5; 61.5]	55.5 [50.8; 60.3]	0.07
**BMI**	26.6 [24.8; 30.7]	24.7 [23.4; 27.2]	27.8 [23.9; 30.9]	0.05
**Total bacterial mass**	13.0 [12.8; 13.5]	12.9 [12.7; 13.3]	12.9 [12.6; 13.3]	0.21
***Lactobacillus* spp.**	6.70 [6.48; 7.60]	7.00 [6.54; 7.92]	6.70 [6.00; 7.12]	0.15
***Bifidobacterium* spp.**	11.0 [10.3; 11.6]	10.9 [10.1; 11.8]	11.0 [10.8; 11.5]	0.91
Escherichia coli	8.95 [8.00; 9.70]	9.30 [8.54; 9.84]	8.74 [7.92; 9.15]	0.17
***Bacteroides* spp.**	12.8 [12.5; 13.3]	12.7 [12.4; 13.2]	12.7 [12.5; 13.0]	0.56

	N (N=41)	SubI (N=31)	I (N=24)	p-value
Faecalibacterium prausnitzii	11.9 [11.7; 12.3]	12.0 [11.7; 12.4]	12.0 [11.7; 12.3]	0.87
Bacteroides thetaomicron	9.30 [0; 10.3]	7.90 [0; 9.45]	8.30 [6.43; 9.30]	0.40
Akkermansia muciniphila	9.90 [8.48; 11.0]	10.3 [7.30; 11.4]	9.39 [4.20; 11.1]	0.75
***Enterococcus* spp.**	0 [0; 5.00]	0 [0; 6.00]	0 [0; 1.25]	0.26
***Escherichia coli* enteropathogenic**	0 [0; 0]	0 [0; 0]	0 [0; 0]	0.35
Klebsiella pneumoniae	0 [0; 0]	0 [0; 0]	0 [0; 0]	0.12
Klebsiella oxytoca	0 [0; 0]	0 [0; 0]	0 [0; 0]	0.33
***Candida* spp.**	0 [0; 0]	0 [0; 0]	0 [0; 0]	0.72
Staphylococcus aureus	0 [0; 0]	0 [0; 0]	0 [0; 0]	0.67
Clostridium difficile	0 [0; 0]	0 [0; 0]	0 [0; 0]	-
Clostridium perfringens	**0 [0; 0]**	0 [0; 6.13]	**2.65 [0; 6.97]**	0.01
Proteus vulgaris mirabilis	0 [0; 0]	0 [0; 0]	0 [0; 0]	0.90
***Citrobacter* spp.**	0 [0; 0]	0 [0; 0]	0 [0; 0]	0.39
***Enterobacter* spp.**	0 [0; 7.70]	5.60 [0; 8.50]	0 [0; 6.13]	0.48
Fusobacterium nucleatum	0 [0; 5.60]	0 [0; 0]	0 [0; 1.25]	0.38
Parvimonas micra	0 [0; 6.48]	5.70 [0; 6.78]	0 [0; 0]	0.05
***Salmonella* spp.**	0 [0; 0]	0 [0; 0]	0 [0; 0]	-
***Shigella* spp.**	0 [0; 0]	0 [0; 0]	0 [0; 0]	0.26
***Blautia* spp.**	8.30 [0; 9.30]	8.48 [0; 9.74]	8.04 [0; 9.77]	0.93
***Acinetobacter* spp.**	7.30 [7.00; 7.60]	7.30 [7.00; 7.81]	7.30 [6.36; 7.60]	0.62
***Streptococcus* spp.**	7.78 [6.70; 8.95]	7.48 [6.84; 8.50]	6.65 [6.00; 8.48]	0.13
Eubacterium rectale	11.0 [10.5; 11.5]	10.8 [10.1; 11.5]	10.8 [10.4; 11.3]	0.75
Roseburia inulinivorans	10.9 [10.3; 11.5]	10.8 [9.69; 11.3]	10.7 [9.78; 11.1]	0.54
***Prevotella* spp.**	9.30 [6.60; 11.3]	9.48 [8.15; 12.0]	10.4 [7.48; 12.0]	0.44
Methanobrevibacter smithii	0 [0; 8.95]	0 [0; 9.00]	6.95 [0; 8.08]	0.92
Methanosphaera stadmanae	0 [0; 5.30]	0 [0; 6.15]	0 [0; 6.35]	0.56
***Ruminococcus* spp.**	8.00 [7.00; 9.00]	6.90 [2.85; 8.00]	7.98 [7.23; 9.00]	0.03

	N (N=41)	SubI (N=31)	I (N=24)	p-value
**GSTP1**	9.25 [9.06; 9.47]	9.36 [9.21; 9.52]	9.22 [8.95; 9.32]	0.05
**GSH**	95.7 [92.1; 99.4]	97.0 [94.9; 99.0]	96.6 [92.9; 98.2]	0.44

N is a control group; SubI – subclinical insomnia group; I – clinical insomnia group including severe insomnia cases. Between-group differences are shown in bold (p < 0.017).

Table S5. Characteristics of ESS groups

	N (N=79)	S (N=17)	p-value (Wilcoxon)
**Age**	57.0 [51.5; 63.0]	56.0 [51.0; 59.0]	0.22
**BMI**	26.0 [23.5; 28.1]	28.4 [26.0; 31.8]	0.07
**Total bacterial mass**	12.9 [12.7; 13.3]	13.3 [13.0; 13.5]	0.16
***Lactobacillus* spp.**	6.85 [6.48; 7.60]	7.00 [6.30; 7.85]	0.52
***Bifidobacterium* spp.**	**11.0 [10.2; 11.6]**	**11.5 [11.0; 11.8]**	**0.04**
Escherichia coli	9.00 [8.30; 9.74]	8.90 [8.60; 9.60]	0.84
***Bacteroides* spp.**	12.7 [12.5; 13.0]	13.0 [12.6; 13.5]	0.08
Faecalibacterium prausnitzii	12.0 [11.7; 12.3]	12.0 [12.0; 12.5]	0.15
Bacteroides thetaomicron	8.30 [0; 9.69]	8.78 [0; 9.78]	0.70
Akkermansia muciniphila	9.48 [6.00; 11.2]	9.90 [7.48; 11.5]	0.59
***Enterococcus* spp.**	0 [0; 5.00]	0 [0; 6.30]	0.07
***Escherichia coli* enteropathogenic**	0 [0; 0]	0 [0; 0]	0.66
Klebsiella pneumoniae	0 [0; 0]	0 [0; 0]	0.42
Klebsiella oxytoca	0 [0; 0]	0 [0; 0]	0.72
***Candida* spp.**	0 [0; 0]	0 [0; 0]	0.44
Staphylococcus aureus	0 [0; 0]	0 [0; 5.30]	0.11
Clostridium difficile	0 [0; 0]	0 [0; 0]	-
Clostridium perfringens	0 [0; 5.50]	0 [0; 5.70]	0.57
Proteus vulgaris mirabilis	0 [0; 0]	0 [0; 0]	0.84

	N (N=79)	S (N=17)	p-value (Wilcoxon)
***Citrobacter* spp.**	0 [0; 0]	0 [0; 0]	0.91
***Enterobacter* spp.**	0 [0; 7.35]	7.00 [0; 7.30]	0.30
Fusobacterium nucleatum	0 [0; 0]	0 [0; 5.00]	0.77
Parvimonas micra	0 [0; 6.15]	6.00 [0; 6.95]	0.07
***Salmonella* spp.**	0 [0; 0]	0 [0; 0]	-
***Shigella* spp.**	0 [0; 0]	0 [0; 0]	0.47
***Blautia* spp.**	8.60 [0; 9.65]	0 [0; 9.85]	0.16
***Acinetobacter* spp.**	7.30 [6.95; 7.60]	7.48 [7.30; 7.78]	0.19
***Streptococcus* spp.**	7.60 [6.65; 8.59]	7.90 [6.00; 8.78]	0.92
Eubacterium rectale	**10.8 [10.0; 11.5]**	**11.3 [10.7; 11.8]**	**0.04**
Roseburia inulinivorans	10.8 [10.0; 11.3]	10.9 [10.0; 11.3]	1.00
***Prevotella* spp.**	**9.30 [7.30; 11.3]**	**11.5 [9.48; 12.0]**	**0.02**
Methanobrevibacter smithii	6.60 [0; 8.93]	0 [0; 9.00]	0.57
Methanosphaera stadmanae	0 [0; 5.39]	0 [0; 7.85]	0.16
***Ruminococcus* spp.**	7.85 [6.48; 8.98]	7.60 [6.48; 9.00]	0.86
**GSTP1**	9.31 [9.08; 9.49]	9.24 [9.05; 9.34]	0.30
**GSH**	96.6 [93.7; 99.4]	97.9 [94.6; 98.4]	0.68

N is a control group; S is a group with the excessive daytime sleepiness. Between-group differences are shown in bold ($p < 0.05$).
